# Genomic epidemiology of *Helicobacter pylori* in regions with high and low risk of gastric cancer, Colombia

**DOI:** 10.3389/fmicb.2025.1742406

**Published:** 2026-02-11

**Authors:** Kevin Guzman, Danilo Igua, Harold Mauricio Casas Cruz, Alvaro Pazos, Arsenio Hidalgo

**Affiliations:** 1 Public Health Research Group, CESUN, University of Nariño, Pasto, Colombia; 2 GICIENSA Group, Medicine Program, University of Nariño, Pasto, Colombia; 3 Department of Biology, Faculty of Exact and Natural Sciences, University of Nariño, Pasto, Colombia; 4 Department of Mathematics and Statistics, Faculty of Exact and Natural Sciences, University of Nariño, Pasto, Colombia

**Keywords:** Colombia, epidemiology, gastric cancer, *H. pylori*, molecular evolution, virulence genes

## Abstract

**Background:**

*Helicobacter pylori* infects more than half of the world’s population and is the main risk factor for gastric cancer, although only a small percentage of those infected develop the disease. This disparity suggests the influence of bacterial, environmental, and host susceptibility factors. In Colombia, the department of Nariño presents a unique scenario: in both the Andean region and the Pacific coast, the prevalence of infection reaches 90%, but gastric cancer rates differ markedly (150/100,000 and 6/100,000 inhabitants, respectively), a phenomenon known as the “Colombian enigma.”

**Methods:**

This study analyzed gastric cancer mortality in 64 municipalities in Nariño, Colombia, using official epidemiological data and genome-based *Helicobacter pylori cagA* and *vacA* evolution and virulence.

**Results:**

The results showed a positive correlation between altitude and gastric cancer mortality. Phylogenomically, two local subpopulations were identified: hspColombia_Andes, predominant in high-risk areas and hspColombia_PacificCoast, associated with low risk. These populations showed genetic overlap, reflecting flow between nearby regions.

**Conclusions:**

Our findings show that the genetic diversity of *Helicobacter pylori*, particularly the hspColombia_Andes and hspColombia_PacificCoast subpopulations, is associated with regional differences in gastric cancer mortality. Furthermore, the influence of environmental factors such as altitude and the association of the *vacA* and *cagA* oncogenes with gastric lesions reinforce their role in pathogenesis and in the possible prediction of cancer risk.

## Introduction

*Helicobacter pylori* (*H. pylori*) is a gram-negative bacterium that infects more than half of the world’s population, with an estimated 4.4 billion people carrying it, although its prevalence in adults has been declining in recent decades (Chen Y. C. et al., 2024; Bashir and Khan, 2023) The infection is mainly acquired in childhood and persists throughout life if it is left untreated, contributing to chronic gastric pathologies and increasing the risk of precursor lesions and gastric cancer (Borka Balas et al., 2022). The discovery of *H. pylori* and its causal relationship with chronic gastritis and peptic ulcers was made in the 1980s by Robin Warren and Barry Marshall. For this finding, which revolutionized the treatment and understanding of *H. pylori* infection and stomach cancer, they received the Nobel Prize in Medicine in 2005 (Marshall, 2008; Otero-Regino, 2022).

*H. pylori* infection is considered the main risk factor for the development of gastric cancer, although the presence of the bacterium is necessary but not sufficient for this process to occur: only between 1 and 3% of infected individuals develop gastric cancer during their lifetime (Guzmán and Pazos, 2023; Ishaq and Nunn, 2015; Mbulaiteye et al., 2009). This low progression rate suggests the influence of dietary and environmental factors and the genetic susceptibility of the host (Acosta-Astaiza et al., 2023).

*H. pylori* has virulence genes, notably *vacA* and *cagA*, which have been directly associated with the pathogenesis and progression of gastric damage. The *cagA* gene encodes an oncoprotein that, interferes with multiple cellular regulatory pathways and causes morphological and functional changes in gastric epithelial cells, facilitating neoplastic processes (Acosta-Astaiza et al., 2023; Martínez Leyva et al., 2021; Salvatori et al., 2023). *vacA* produces a cytotoxin that causes cell vacuolization, apoptosis, and alters epithelial homeostasis. Some allelic variants, such as s1/m1, are linked to greater tissue damage and oncological risk (Acosta-Astaiza et al., 2023; Martínez Leyva et al., 2021; Salvatori et al., 2023). The interaction of these genetic factors (*vacA* and *cagA*) with the chronic inflammatory response promotes a sequence of lesions that begins with chronic gastritis, atrophy, intestinal metaplasia, dysplasia, and finally gastric adenocarcinoma (Piazuelo and Correa, 2013).

Currently, *H. pylori* is classified into eight main modern populations, based on their geographic distribution and genetic diversity: hpAfrica1, hpAfrica2, hpNEAfrica, hpEurope, hpEastAsia, hpNorthAsia, hpAsia2, and hpSahul (Moodley and Linz, 2009; Moodley et al., 2021; Yamaoka et al., 2023; Guzman K. A. et al., 2024). These populations have specific subpopulations that reflect evolutionary and migratory patterns of human populations (Moodley and Linz, 2009; Moodley et al., 2021; Yamaoka et al., 2023; Guzman K. A. et al., 2024). This phylogeographic diversity is key to understanding the evolution and adaptability of *H. pylori*, as well as its differential impact on gastric pathogenesis among different populations.

Understanding the genetic diversity of *H. pylori* and its populations is key to explaining the differences in risk in diverse regions. In this regard, the department of Nariño represents a particular case in Colombia where, despite a high prevalence of *H. pylori* infection in its Andean and Pacific regions (90%), the incidence of gastric cancer varies considerably between the two areas. This disparity, with high incidence rates in the Andean region (150/100,000 inhabitants) and low rates on the Pacific coast (6/100,000 inhabitants), known as the “Colombian enigma,” is an ideal model for studying the interaction between genetic and environmental bacterial factors that modulate disease risk (Guzmán and Pazos, 2023; Piazuelo and Correa, 2013). Therefore, identifying the genetic characteristics of local *H. pylori* populations, together with epidemiological analysis, can provide key information for the development of prevention, detection, and treatment strategies tailored to the specific needs of these populations.

## Materials and methods

### Mortality data

To estimate the mortality rate from gastric cancer in the department of Nariño, data provided by the Nariño Departmental Health Institute^1^ were collected and analyzed. These data included official mortality records associated with gastric cancer in the 64 municipalities of the department corresponding to the period 2011–2020.

The altitude data were obtained through the national cartography application provided by the Agustín Codazzi Geographical Institute (IGAC).^2^ Subsequently, a mortality rate map was created for the entire department using ArcGIS v3.0 software. Mortality data were georeferenced by municipality, allowing for the visualization of the spatial distribution of gastric cancer mortality in Nariño. This cartographic approach facilitated the identification of areas with higher and lower mortality rates, contributing to a detailed regional analysis.

### Obtaining bioinformatic data

The initial bioinformatic data used in this study come from complete *H. pylori* genomes sequenced in different regions of the world, which are available in PubMLST. To identify housekeeping genes, sequences annotated in the PubMLST database^3^ (Jolley et al., 2018) were used. A total of 67 isolates were used for the department, 48 genomes from municipalities with a high risk of gastric cancer, while for the Pacific coast in the municipalities of Tumaco and Barbacoas, 19 *H. pylori* isolates were used (Table 1).

**TABLE 1:** *Helicobacter pylori* isolates analyzed from two regions with different gastric cancer risks.

Isolated	Risk of gastric cancer	Municipality	Isolated	Risk of gastric cancer	Municipality
SV328_2	High risk	Tuquerres	CR004	High risk	Pasto
SV340_2	High risk	Tuquerres	CR005	High risk	Pasto
SV355_2	High risk	Tuquerres	CR031	High risk	Pasto
SV376_1	High risk	Tuquerres	CR045	High risk	Pasto
SV380_1	High risk	Tuquerres	CR047	High risk	Pasto
SV397_2	High risk	Tuquerres	CR048	High risk	Pasto
SV449_1	High risk	Tuquerres	CR054	High risk	Pasto
PZ5056	High risk	Tuquerres	AP029	High risk	Florida
PZ5080	High risk	Tuquerres	CR44	High risk	Pasto
PZ5086	High risk	Tuquerres	CR45	High risk	Pasto
HpGP-COL-310	High risk	Florida	CR56	High risk	Pasto
HpGP-COL-318	High risk	Florida	CR60	High risk	Pasto
CR12	High risk	Florida	CR71	High risk	Pasto
NQ1671	High risk	Nariño	CR46	High risk	Samaniego
NQ1701	High risk	Nariño	PZ5005_3A3	Low risk	Tumaco
NQ352	High risk	Nariño	PZ5006_3A3	Low risk	Tumaco
NQ4191	High risk	Nariño	PZ5009_3A2	Low risk	Tumaco
NQ4228	High risk	Nariño	PZ5016_3A3	Low risk	Tumaco
NQ4216	High risk	Nariño	PZ5019_3A3	Low risk	Tumaco
NQ4200	High risk	Nariño	PZ5033_3A2	Low risk	Tumaco
NQ4161	High risk	Nariño	PZ5004	Low risk	Tumaco
NQ4110	High risk	Nariño	PZ5024	Low risk	Tumaco
NQ4099	High risk	Nariño	PZ5026	Low risk	Tumaco
NQ4076	High risk	Nariño	MT5105	Low risk	Tumaco
NQ4053	High risk	Nariño	MT5111	Low risk	Tumaco
NQ4044	High risk	Nariño	MT5114	Low risk	Tumaco
AP002	High risk	Florida	MT5118	Low risk	Tumaco
AP015	High risk	Florida	MT5119	Low risk	Tumaco
AP018	High risk	Florida	MT5124	Low risk	Tumaco
AP021	High risk	Florida	MT5125	Low risk	Tumaco
AP022	High risk	Florida	MT5135	Low risk	Tumaco
AP025	High risk	Florida	MT5136	Low risk	Tumaco
AP028	High risk	Florida	CR41	Low risk	Barbacoas
AP031	High risk	Florida			

### Multilocus sequence typing analysis

To identify the housekeeping genes *atpA, efp, mutY, ppa, trpC, ureI*, and *yphC* in the genomes, annotation was performed using the PubMLST database (see text footnote 3; Jolley et al., 2018). The concatenated sequences were aligned with Muscle (Edgar, 2004) and the phylogenetic analysis was constructed using a Neighbor-joining method, based on a T92 + G + I evolutionary model in MEGA 12 (Kumar et al., 2024). A bootstrap analysis with 1,000 replicates was performed to assess the robustness of the phylogenetic tree, which was subsequently visualized and edited in iTol^4^ (Letunic and Bork, 2024).

### Phylogenomic analysis based on the core genome of *Helicobacter pylori*

All complete genome sequences were imported into the BIGSdb database (Jolley et al., 2018), which is dedicated to isolated bacterial genome sequences. Subsequently, a gene-by-gene alignment was performed using the CDS sequences of *H. pylori* strain 26695 as a reference, and the alignments were exported from the database. The matrix obtained from the genome comparator generated by BIGSdb was used to construct the phylogenetic tree using the maximum likelihood method with the GTR + G + I model in the IQ-Tree software (Nguyen et al., 2015). The resulting phylogenetic tree was visualized and edited in iTol (see text footnote 4; Letunic and Bork, 2024).

### Genotyping of the *cagA* and *vacA* oncogenes of *Helicobacter pylori* from the department of Nariño

Of the 67 previously used *H. pylori* genomes, 60 complete genomes available for Nariño were downloaded from the PubMLST database (see text footnote 3) because they contained complete histopathology metadata. Of the 60 isolates, 41 originated from municipalities in the Andean region of Nariño a high gastric cancer risk area (Pasto, Túquerres and La Florida) and 19 from municipalities in the Pacific region, a low gastric cancer risk area (Tumaco and Barbacoas). Isolates were categorized according to histopathological diagnosis into two groups: of the 41 isolates from the Andean region, 20 were from patients with non-atrophic gastritis (NAG) and 21 from patients with atrophic gastritis (AG); of the 19 isolates from the Pacific region, 10 were from patients with NAG and 9 from patients with AG, yielding a total of 30 patients for each histological diagnosis.

Reference sequences for the virulence genes *cagA* and *vacA* were obtained from the consolidated annotations in the Virulence Factor Database (VFDB),^5^ using *H. pylori* strain 26695 as the reference genome (CagA: WP_000180747.1; VacA: WP_000405496.1) (Hatakeyama, 2017; Zhou et al., 2025). Initially, the presence or absence of each gene was assessed individually using the BLAST tool integrated in PubMLST. BLAST was run with default parameters; a gene was considered present when the alignment showed ≥ 90% identity and ≥ 85% coverage relative to the 26695 reference (Muñoz et al., 2025). For *cagA*-positive strains, sequences were exported in FASTA format, translated to amino acids, open reading frame (ORF) integrity was checked, and alignments were performed in MEGA 12 to locate EPIYA motifs (Kumar et al., 2024), using the amino-acid sequences presented in Table 2 for strain 26695 as reference (Rodríguez Gómez et al., 2020). For *vacA-*positive strains, allele typing was performed using the *in silico* PCR option integrated in PubMLST by entering the primers described in Table 2.

**TABLE 2:** Reference amino acid sequence for each EPIYA motif of *cagA* and primers used for typing *vacA* alleles of *Helicobacter pylori*.

Region	Motif/Primers	Primer sequence or amino acid sequence (5’–3’)	Size	References
*cagA*	EPIYA-A	EPIYAKVNKKKAGQ	14 aa	(Rodríguez Gómez et al., 2020)
	EPIYA-B	EPIY (A/T) QVAKKVNAKI	15 aa	
	EPIYA-C	EPIYATIDDLGGP	13 aa	
	EPIYA-D	EPIYATIDFDEANQAG	16 aa	
*vacA s1a*	Forward	CTCTCGCTTTAGTAGGAGC	213 pb	(Yamazaki et al., 2005)
	Reverse	CTGCTTGAATGCGCCAAAC		
*vacA s1b*	Forward	AGCGCCATACCGCAAGAG	187pb	
	Reverse	CTGCTTGAATGCGCCAAAC		
*vacA s2*	Forward	GCTAACACGCCAAATGATCC	199 pb	
	Reverse	CTGCTTGAATGCGCCAAAC		
*vacA m1*	Forward	GGTCAAAATGCGGTCATGG	290 pb	(Yamazaki et al., 2005; Atherton, 1998)
	Reverse	CCATTGGTACCTGTAGAAAC		
*vacA m2*	Forward	GGAGCCCCAGGAAACATTG	352 pb	
	Reverse	CATAACTAGCGCCTTGCAC		
*vacA i1*	Forward	GTTGGGATTGGGGGAATGCCG	426 pb	(Rhead et al., 2007)
	Reverse	TTAATTTAACGCTGTTTGAAG		
*vacA i2*	Forward	GTTGGGATTGGGGGAATGCCG	432 pb	
	Reverse	GATCAACGCTCTGATTTGA		

### Statistical analysis

Statistical analysis was performed in RStudio v. 4.1.1. For the correlation analysis, given that the mortality data did not show a normal distribution (Kolmogorov-Smirnov test), correlations were performed for height above sea level and mortality using Spearman’s non-parametric coefficient, taking a value of *p* < 0.05 as statistically significant. To evaluate the relationship between the distribution of *H. pylori* strains and risk based on gastric cancer mortality data (high vs. low) for the municipalities studied, Fisher’s exact test was applied, with *p* < 0.05 as significant. In the analyses for the *vacA* and *cagA* oncogenes, chi-square tests were applied when the expected frequencies were ≥ 5 and Fisher’s exact test in cases with lower counts. Odds ratios (OR) and 95% confidence.

## Results

**FIGURE 1:**
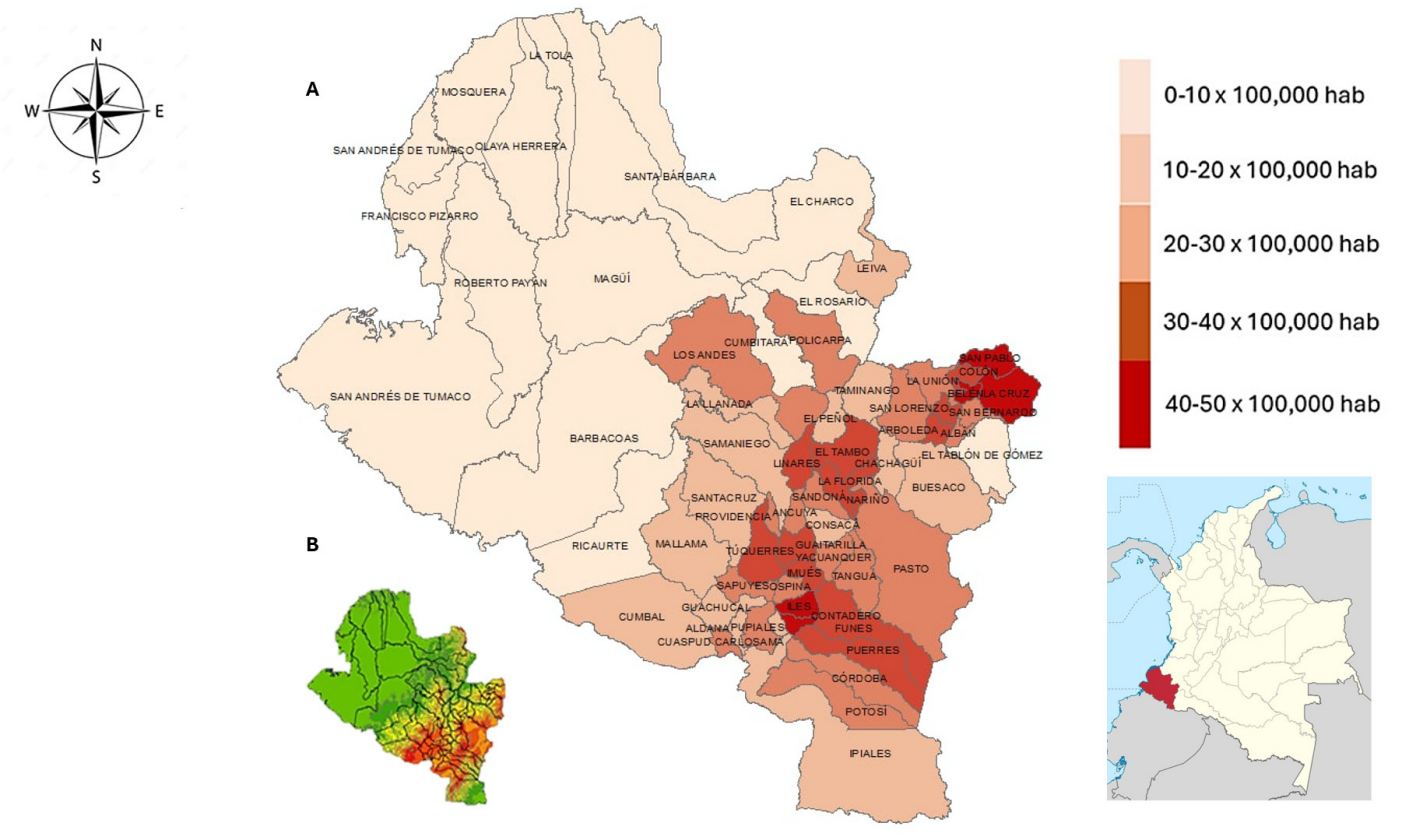
Spatial distribution of mortality and altitude across the 64 municipalities of the department of Nariño, Colombia. **(A)** Mortality map showing lower values in municipalities along the Pacific coast (0-10 per 100,000 inhabitants) and higher values in municipalities located in the Andean region (10-50 per 100,000 inhabitants). **(B)** Altitude map of the municipalities, where green areas represent locations at sea level, and colors ranging from yellow to red indicate municipalities situated at higher elevations above sea level.

Mortality rates were high in municipalities in the Andean region, ranging from 20 to 50 per 100,000 inhabitants. In contrast, municipalities on the Pacific coast had considerably lower mortality rates, ranging from 0 to 10 per 100,000 inhabitants, as shown in Figures 1A,B illustrates the altitude of the municipalities: the areas in green correspond to those located at sea level, while the colors ranging from yellow to red represent municipalities with greater altitude above sea level (Figure 1).

**FIGURE 2:**
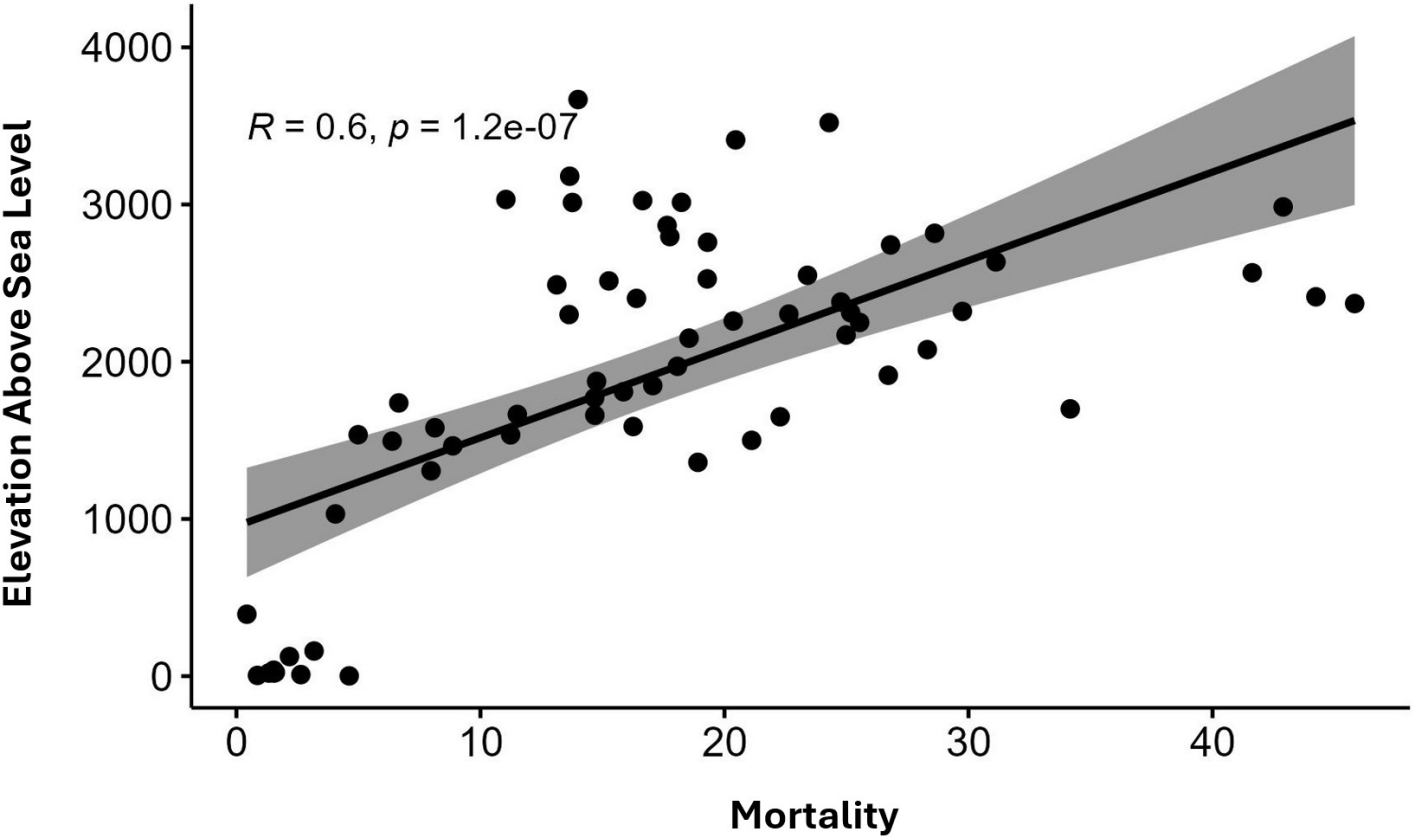
Correlation analysis between altitude on the y-axis and mortality on the x-axis in the 64 municipalities of the department of Nariño, Colombia.

The relationship between altitude above sea level and mortality rates in the 64 municipalities of the department of Nariño was analyzed. Spearman’s correlation analysis yielded a *p* < 0.05, establishing a direct correlation between altitude and mortality from gastric cancer in the department of Nariño. The finding indicates that, in the department of Nariño, the higher the altitude above sea level, the higher the mortality rate from gastric cancer. This suggests that altitude is acting as a factor associated with the risk of developing stomach cancer (Figure 2).

**FIGURE 3:**
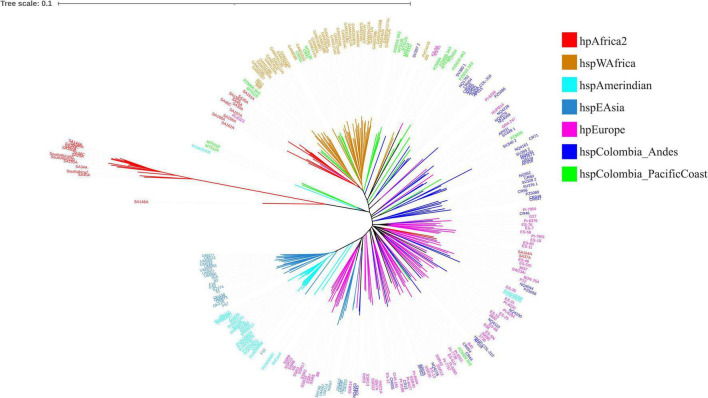
MLST phylogenetic tree of *Helicobacter pylori* isolated from the department of Nariño with isolates from other regions of the world.

When performing MLST analysis on *H. pylori* isolates from the department of Nariño, the formation of clusters corresponding to the hpAfrica2, hspWAfrica, hspAmerindian, and hspEAsia strains was observed, while isolates of European origin were grouped with both strains from the Andean region and those from the Pacific coast. Interestingly, some isolates from the Pacific coast grouped specifically with strains from West Africa. These findings demonstrate the European and African migration process that occurred 500 years ago to the American continent, bringing with it this genetic load in microorganisms, especially the hpEurope and hspWAfrica strains (Figure 3).

**FIGURE 4:**
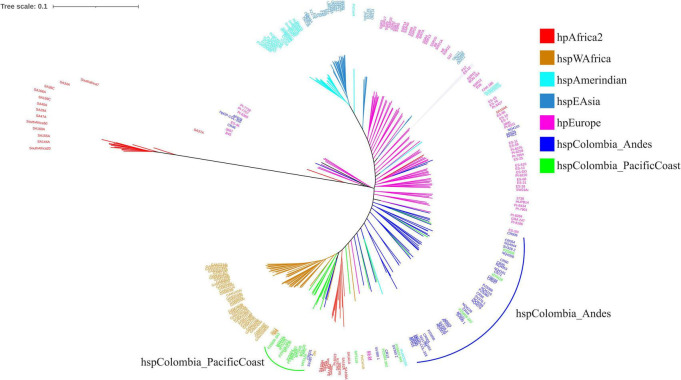
Phylogenomic tree of *Helicobacter pylori* isolates obtained from the Andean region and Pacific coast of the department of Nariño, Colombia.

When analyzing the phylogenomic tree, it was observed that the lineages presented a more defined grouping. The most ancestral group, which formed the root of the tree, corresponded to hpAfrica2, and close to this, European isolates and isolates from the Andean region of Nariño were identified, suggesting the preservation of an ancestral homology with these strains. Unlike what was evidenced by MLST, in this tree the hspAmerindian isolates were clearly differentiated from the Asian isolates. Likewise, in contrast to the MLST results, the isolates from the Andean region formed an independent lineage called hspColombia_Andes, while on the Pacific coast another distinct lineage was identified, called hspColombia_PacificCoast, which differed from the hspWAfrica isolates. In both lineages, isolates from both the Andean region and the Pacific coast were also observed to overlap, suggesting the existence of gene flow between these populations, which are only 150 km apart (Figure 4).

**FIGURE 5:**
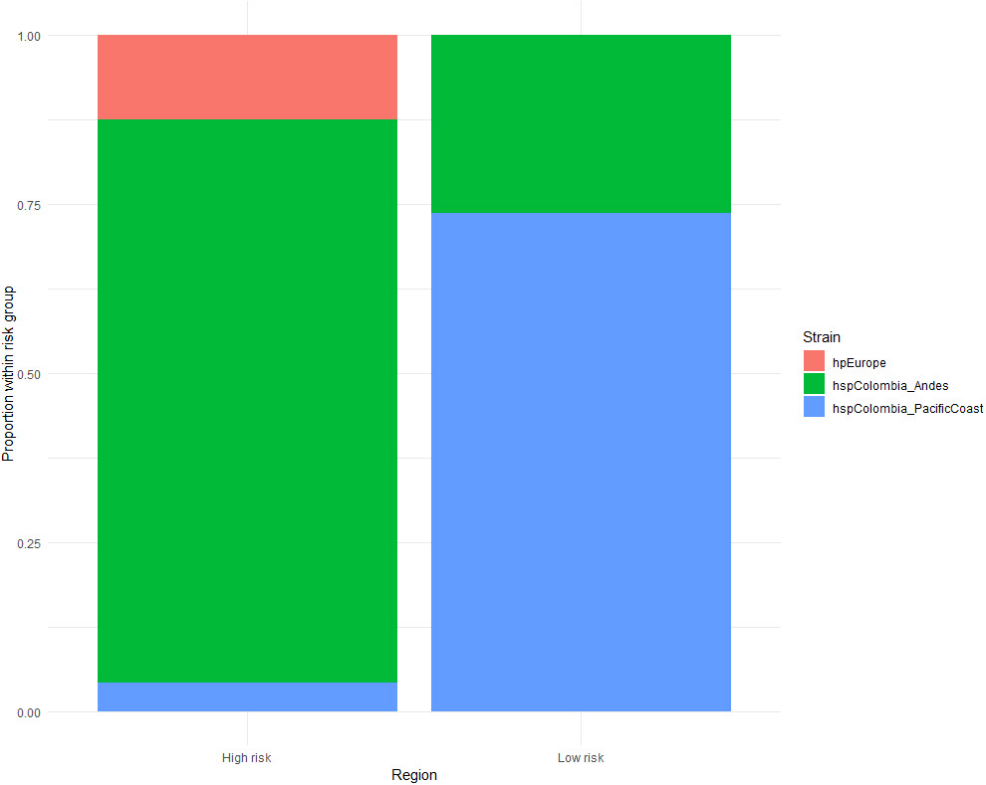
Relative frequencies of *Helicobacter pylori* strains identified in the regions studied. Each bar corresponds to a geographical region in the Andes and Pacific Coast, while the colors indicate the proportional distribution of the different lineages.

In the Andean region, classified as high risk, the hspColombia_Andes population predominated (*n* = 40), with a lower representation of hpEurope strains (*n* = 6) and a marginal proportion of hspColombia_PacificCoast (*n* = 2). In contrast, in the Pacific coastal region, characterized by low risk, most isolates belonged to the hspColombia_PacificCoast population (*n* = 14), while the frequency of hspColombia_Andes strains (*n* = 5) was low and hpEurope was practically absent. Statistical analysis using Fisher’s exact test showed a significant association between strain distribution and geographical risk of gastric cancer (*p* < 0.05), suggesting that bacterial population structure plays a decisive role in the epidemiological differences observed between the two regions (Figure 5).

**FIGURE 6:**
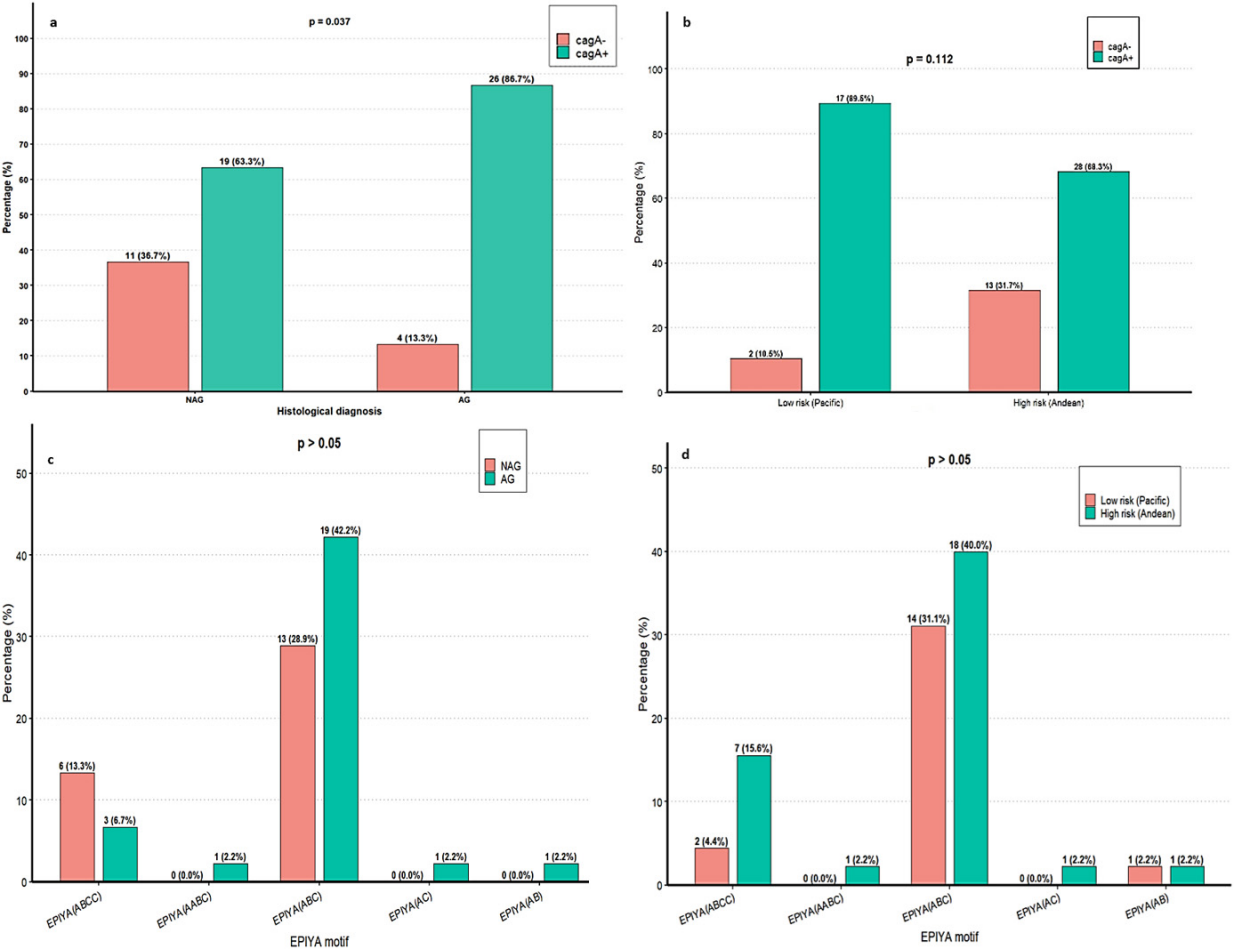
Association of *cagA* and EPIYA motifs with histological diagnosis and geographic region. **(a)** Presence of *cagA* by histological diagnosis. (**b**) Presence of *cagA* by region. **(c)** Distribution of EPIYA motifs by histological diagnosis. **(d)** Distribution of EPIYA motifs by region.

In the analysis of *vacA* and *cagA*, *cagA* was detected in 45 strains overall (75%; 45/60). Regarding histopathological diagnosis, *cagA* was detected in 86.7% (26/30) of strains isolated from AG, compared with 63.3% (19/30) of strains from NAG (Figure 6a). This association was statistically significant (p = 0.037; OR = 3.76; 95% CI, 1.04–13.60), indicating that the odds of strains from AG being *cagA*-positive were 3.76 times the odds for strains from NAG. By region, *cagA* prevalence was higher in the low-risk region (89.5%; 17/19) than in the high-risk region (68.3%; 28/41), although this difference did not reach statistical significance (Figure 6b).

In the 45 *cagA*-positive strains, the EPIYA (ABC) pattern was the most frequent in both groups: 42.2% in AG (19/45) and 28.9% in NAG (13/45), with no significant differences (Figure 6c). At the regional level, this pattern also predominated in both the low-risk region (31.1%; 14/45) and the high-risk region (40.0%; 18/45), with no statistical significance (Figure 6d). Although no statistically relevant differences were identified, the distribution suggests possible variations according to lesion type and region.

**FIGURE 7:**
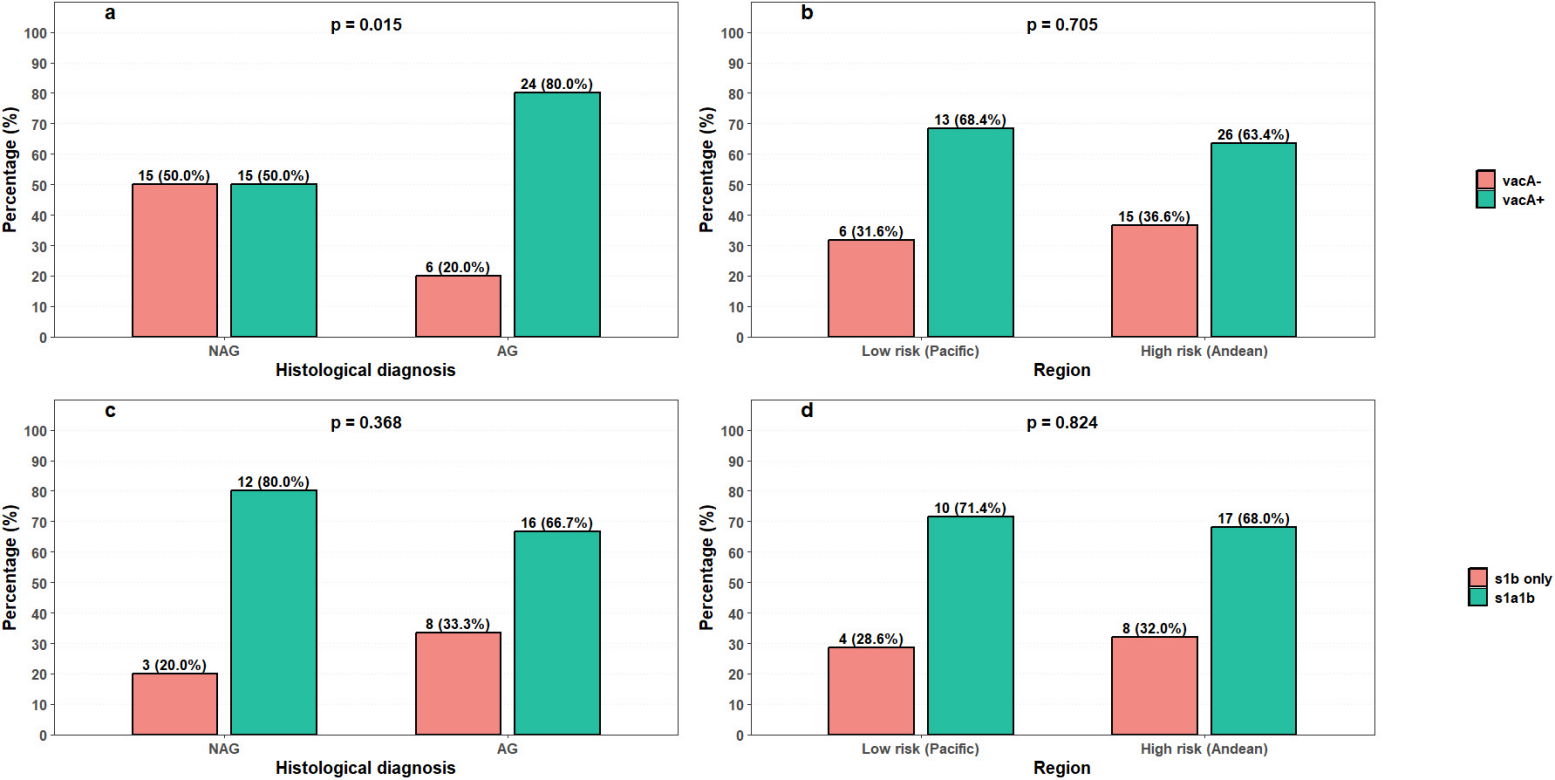
Association of the *vacA* gene and its allelic variants with histological diagnosis and geographic region. **(a)** Presence of *vacA* by histological diagnosis. **(b)** Presence of *vacA* by region. **(c)** Distribution of *vacA* alleles by histological diagnosis. **(d)** Distribution of *vacA* alleles by region.

The *vacA* gene was detected in 39 strains overall (65%; 39/60). In strains associated with AG, *vacA* was detected in 80.0% of isolates (24/30), compared with 50.0% (15/30) in NAG. This association was statistically significant (*p* = 0.015; OR = 4.0; 95% CI, 1.27–12.6), indicating that the odds of strains from AG being *vacA*-positive were 4.0 times the odds for strains from NAG (Figure 7a). By region, *vacA* was detected in 68.4% (13/19) of strains from low-risk areas and 63.4% (26/41) of strains from high-risk areas, with no significant association (Figure 7b).

In allele characterization of the *vacA*-positive strains, the m1 and i1 regions were detected in all isolates, while the s1a1b combination was observed in 66.7% (16/24) of strains from AG and 80.0% (12/15) of strains from NAG, with no significant differences (Figure 7c). Similarly, the s1a1b allele combination was more frequent in the low-risk region (71.4%; 10/14) than in the high-risk region (68.0%; 17/25), although without statistical differences (Figure 7d).

## Discussion

*H. pylori* is one of the most ancient microorganisms associated with *Homo sapiens.* Its infection is estimated to have occurred approximately 130,000 years ago, and for the past 60,000 years, it has accompanied humans from East Africa, giving rise to different bacterial populations in each settlement (Moodley et al., 2012; Linz et al., 2007). Only about 500 years ago, the arrival of Europeans and Africans to the American continent introduced a new set of pathogens, including *H. pylori*. The encounter between the hspAmerindian, hpEurope, hpAfrica2, and hspWAfrica strains gave rise to new, independent lineages of *H. pylori* in the Americas (Thorell et al., 2017; Guzman et al., 2023; Guzman K. A. et al., 2024).

The two new *H. pylori* subpopulations suggest that the bacteria are undergoing a constant process of evolution and adaptation to the new gastric environment, the host’s immune response, and the geographical conditions in which these populations are found (Thorell et al., 2017; Guzman et al., 2023; Guzman K. A. et al., 2024). In the Andes region, which has a higher risk, most isolates corresponded to a predominant local population, hspColombia_Andes, while European-origin strains and those typical of the Pacific coast appeared in much smaller proportions. In contrast, in the Pacific region, classified as low risk, the opposite pattern was observed, with a strong representation of the area’s native population (hspColombia_PacificCoast) and a low presence of isolates characteristic of the Andes, while European strains were practically absent. Statistical analysis confirmed that this differential distribution is not random but is significantly associated with the geographical risk of gastric cancer (*p* < 0.05). Likewise, the finding of strains with genetic overlap in both areas suggests the existence of population exchange facilitated by geographical proximity, which maintains a dynamic and diverse gene pool within the species.

These results contrast with previous MLST studies, which found that strains from the Pacific coast were of African and European origin, while in the Andean region, particularly in Túquerres, the strains were of European origin. This coevolutionary mismatch was identified as the cause of gastric lesions and gastric cancer in the Andean region, whereas on the Pacific coast, the explanation for the low incidence and mortality from gastric cancer was related to the “African enigma,” associated with the host–pathogen coevolutionary process (de Sablet et al., 2011; Kodaman et al., 2014).

The remarkable difference in gastric cancer mortality rates between the Andean region (high mortality) and the Pacific coast (low mortality), despite the high prevalence of infection in both areas (90%) (Guzmán and Pazos, 2023), confirms the well-known “Colombian enigma.” This disparity reinforces the hypothesis that the mere presence of *H. pylori* is not sufficient to explain cancer development and that environmental factors such as altitude play an important role, as evidenced by the statistically significant correlation between altitude and mortality. Altitude may influence physiological mechanisms, exposure to different environmental factors, or lifestyle patterns that, in turn, modulate cancer risk.

Moreover, the analyses show that *cagA* and *vacA* are significantly associated with atrophic gastritis compared to non-atrophic gastritis; this finding is consistent with previous studies reporting an association between *cagA/vacA* genotypes and preneoplastic lesions in Andean populations (Guzmán J. et al., 2024; Bustos-Fraga et al., 2023; Takahashi-Kanemitsu et al., 2020). The *cagA* oncogene modulates the immune response and innate inflammatory pathways by influencing mitochondrial integrity and the inflammasome. Recent studies have shown that *cagA* can activate the *NLRP3* inflammasome, increasing ROS, *IL-1*β/*IL-18*, and promoting migration and invasion (Chen D. et al., 2024; Zhang et al., 2022). Similarly, in adaptive immunity, *cagA* promotes evasion of effector T cell responses by increasing PD-L1 levels in gastric cell-derived exosomes, inhibiting CD8 + proliferation and function, thereby promoting local tolerance and gastric lesion progression (Wang et al., 2023). However, the relationship between the mere presence of *cagA* and clinical risk is nuanced by that human factors such as ancestry, diet, high salt consumption, and evolutionary lineage with *H. pylori* modulate the likelihood of progression to severe lesions (Guzmán and Pazos, 2023).

For its part, *vacA* induces and dysregulates mitophagy and autophagy, causes alterations in lysosomal trafficking, and can prevent the lysosomal degradation of bacterial and cellular proteins (Wang et al., 2022; Zhu et al., 2017), facilitating the intracellular accumulation of *cagA* and thus amplifying its oncopathogenic effects, a synergistic effect in which it enhances the activity and persistence of *cagA* in epithelial cells, amplifying chronic inflammation and precancerous damage (Abdullah et al., 2019). In addition, it can actively interfere with the maturation and function of antigen-presenting cells or directly with T cells by suppressing *IL-23* and inducing *IL-10*/*TGF-*β, promoting Treg differentiation and a tolerogenic microenvironment that weakens the adaptive response (Altobelli et al., 2019).

In contrast, no significant association was detected between regional origin and the overall frequency of these genes, a finding consistent with studies that found no consistent geographical differences between areas of proven risk in Colombia (Trujillo et al., 2014), although it contrasts with multiple studies reporting that *H. pylori* strains from regions of Colombia with high rates of gastric cancer express higher levels of *cagA* and *vacA* than strains from regions with lower rates of gastric cancer (Bravo et al., 2002; Sicinschi et al., 2010; Loh et al., 2011). The absence of an association between these virulence genes and geographic region may be explained by the phylogenetic overlap between strains classified as hspColombia_Andes and hspColombia_PacificCoast, which is indicative of gene flow across regions. Such admixture is consistent with coinfection or multiple colonization of distinct gastric niches by strains of different ancestry within the same host, a dynamic that can disrupt the expected geographic distribution of these genes (de Sablet et al., 2011; Guzmán and Pazos, 2023). Moreover, host susceptibility factors, particularly polymorphisms or mutations in cytokine genes such as *IL-1B* and *IL-10*, can increase an individual’s predisposition to severe gastric disease irrespective of geographic origin (Camargo et al., 2006).

The molecular characterization of the predominance of the EPIYA-C motif in both strains associated with atrophic gastritis and those from the high-risk region is consistent with recent descriptions of Colombian isolates that document ABC patterns as frequent in the region (Muñoz et al., 2025; Rodríguez Gómez et al., 2020); At the same time, other studies have linked a higher number of EPIYA-C repeats with greater oncogenic capacity in cell assays and with more severe histology (Quiroga et al., 2010), and a recent study in Latin America showed that microvariants in the *cag* pathogenicity island may nuance the association between *cagA* and disease, suggesting that the presence of EPIYA-C alone may not be sufficient to increase risk in the absence of additional repeats (Rizzato et al., 2020).

The presence of the *vacA* m1 and i1 alleles variants associated with increased cytotoxicity in all *vacA*-positive isolates, together with the predominance of the s1a1b combination, yet the absence of a significant association with lesion type or geographic origin, may be explained by high local genetic diversity that facilitates coinfection by strains of distinct ancestry. When combined with *H. pylori*’s elevated rate of homologous recombination via horizontal gene transfer, this diversity can promote the formation of genetic mosaics (mixed s1a1b genotypes) and favor the emergence and persistence of more virulent allele combinations (m1i1), thereby enhancing bacterial fitness. Collectively, these processes may modulate clinical outcomes (Akeel et al., 2019; García-Ortíz et al., 2011; Gutiérrez-Escobar et al., 2019; Wroblewski et al., 2010).

Furthermore, the development of gastric cancer in Colombian populations is determined by a complex interaction between helminth co-infection, immune response, environmental factors, diet, and proximity to volcanoes. *H. pylori* infection induces a proinflammatory Th1-type immune response characterized by the production of cytokines such as *IL-10-1082AG*, which promote inflammation and gastric damag (Piazuelo et al., 2008; Correa, 2013; Torres et al., 2013). However, helminth co-infection can modulate this response, shifting it toward an anti-inflammatory Th2 profile, thereby reducing tissue damage and potentially decreasing cancer risk (Piazuelo et al., 2008). From an environmental perspective, altitude and exposure to volcanic particles in the Andean region generate additional pro-oxidative and inflammatory conditions (Correa, 2013; Torres et al., 2013). Added to this is a diet predominantly based on potatoes and beans, with low consumption of fruits and vegetables, which promotes chronic gastric inflammation (Piazuelo et al., 2008). This scenario contrasts with that of the Pacific coast, where the diet is more varied and rich in fruits, vegetables, and seafood, which is associated with lower gastric cancer mortality (Guzmán and Pazos, 2023; Torres et al., 2013).

This study presents several limitations that should be considered when interpreting the results. First, although 67 complete *Helicobacter pylori* genomes were included, sequence availability depended on public databases, which may have introduced selection bias and limited the representativeness of certain geographic areas. In addition, the number of isolates per municipality was not homogeneous, particularly in low-risk regions, which could affect the robustness of comparative analyses between bacterial subpopulations. The histopathological information used was obtained from records associated with the available genomes, making it impossible to standardize and verify the clinical conditions across all patients.

## Conclusion

*H. pylori* is a continuously evolving microorganism whose coevolution with humans has given rise to new subpopulations, including hspColombia_Andes and hspColombia_PacificCoast. This genetic diversity is associated with the marked difference in gastric cancer mortality between the Andean region and the Pacific coast, reinforcing the role of bacterial population structure.

The pronounced difference in gastric cancer mortality rates between the Andean and Pacific regions, despite the high prevalence of infection in both, supports the so-called “Colombian enigma” and demonstrates that the presence of *H. pylori* alone is not sufficient to explain carcinogenesis. Environmental factors such as altitude play a decisive role in modulating gastric cancer risk.

The analysis of the *vacA* and *cagA* oncogenes confirms their association with the progression of gastric lesions, positioning them as potential genetic biomarkers for predicting progression to more advanced stages and the risk of gastric cancer. Although *vacA* alleles and the EPIYA motifs of the *cagA* gene showed no relationship with lesion severity in this study, the sample size represents a limitation that could be addressed in future research.

## Data Availability

The original contributions presented in this study are included in this article/supplementary material, further inquiries can be directed to the corresponding authors.

## Footnotes

1
https://www.idsn.gov.co/

2
https://www.igac.gov.co/

3
https://pubmlst.org/helicobacter/

4
https://itol.embl.de/

5
https://www.mgc.ac.cn/cgi-bin/VFs/genus.cgi?Genus=Helicobacter
